# Translation stalling induced mitochondrial entrapment of ribosomal quality control related proteins offers cancer cell vulnerability

**DOI:** 10.21203/rs.3.rs-4899860/v1

**Published:** 2024-09-13

**Authors:** Rani ojha, Ishaq Tantray, Shouryarudra Banerjee, Suman Rimal, Sandiya Thirunavukkarasu, Saripella Srikrishna, Wah Chiu, Uttam Mete, Aditya Sharma, Nandita Kakkar, Bingwei Lu

**Affiliations:** Post Graduate Institute of Medical Education and Research; Stanford University; Post Graduate Institute of Medical Education and Research; Stanford University; Post Graduate Institute of Medical Education and Research; Institute of Science, Banaras Hindu University; Stanford University; Post Graduate Institute of Medical Education and Research; Post Graduate Institute of Medical Education and Research; Post Graduate Institute of Medical Education and Research; Stanford University

**Keywords:** Ribosome-associated quality control (RQC), bladder cancer, cancer stem cells (CSCs), ZNF598, ABCE1, translation stalling, mitochondria, stalling-induced mitochondrial stress (SIMS), emetine

## Abstract

Ribosome-associated quality control (RQC) monitors ribosomes for aberrant translation. While the role of RQC in neurodegenerative disease is beginning to be appreciated, its involvement in cancer is understudied. Here, we show a positive correlation between RQC proteins ABCE1 and ZNF598 and high-grade muscle-invasive bladder cancer. Translational stalling by the inhibitor emetine (EME) leads to increased mitochondrial localization of RQC factors including ABCE1, ZNF598, and NEMF, which are continuously imported into mitochondria facilitated by increased mitochondrial membrane potential caused by EME. This reduces the availability of these factors in the cytosol, compromising the effectiveness of RQC in handling stalled ribosomes in the cytosol and those associated with the mitochondrial outer membrane (MOM). Imported RQC factors form aggregates inside the mitochondria in a process we term stalling-induced mitochondrial stress (SIMS). ABCE1 plays a crucial role in maintaining mitochondrial health during SIMS. Notably, cancer stem cells (CSCs) exhibit increased expression of ABCE1 and consequently are more resistant to EME-induced mitochondrial dysfunction. This points to a potential mechanism of drug resistance by CSCs. Our study highlights the significance of mitochondrial entrapment of RQC factors such as ABCE1 in determining the fate of cancer cells versus CSCs. Targeting ABCE1 or other RQC factors in translational inhibition cancer therapy may help overcome drug resistance.

## INTRODUCTION

The translation of mRNAs into proteins governs cellular homeostasis, growth, and survival, impacting diverse physiological processes (1, 2). Protein synthesis is a complex process that can generate faulty proteins with cytotoxic properties (3). Cells have evolved complex ribosome-associated quality control (RQC) mechanisms to clear aberrant translational products that undergo ribosomal stalling (4–6). Due to the emerging and ever-increasing role of protein synthesis in the context of cancer cells, it is quickly becoming imperative to target this mechanism that acts as a reserve of building blocks for multiplying cancer cells (7). A growing body of research suggests that the regulatory mechanisms involved in protein synthesis are important in regulating tumor growth and drug resistance and that targeting these translation processes may serve as a novel therapeutic paradigm for cancer (8). For example, dyshomeostasis of ribosome biogenesis and alterations in the number, size, and shape of nucleoli are now being investigated as potential hallmarks of cancer (9). It is expected that insights into the mechanisms of cancer cell resistance to therapies targeting ribosome biogenesis may bring new perspectives into the molecular basis of cancer susceptibility and new clinical interventions for cancer therapy.

The RQC pathway was initially studied under in vitro conditions or in unicellular yeast (4–6, 10). It is important to understand how this pathway exerts paramount influence on proteins destined for membrane-bound organelles, particularly those co-translationally imported into mitochondria (11, 12). This could provide valuable insights into the cellular functions of the RQC process. In principle, two systems can clear aberrant mitochondrial proteins that are stalled on mitochondrial outer membrane (MOM) associated cytosolic ribosomes: the RQC pathway that acts before their full import into mitochondria and the mitochondrial chaperone and protease networks that act following their import (13, 14). Ribosomes associated with MOM stall in response to mitochondrial stress in a process termed MISTERMINATE (11). Ribosome stalling will cause ribosome collision, which activates the early RQC machinery that mediates the recognition and dissociation of the collided ribosomes, and the subsequent quality control by the late RQC factors of the nascent peptidyl-tRNA associated with the 60S subunit (15). Key factors involved in this process are ZNF598 and RACK1, which recognize the distinct 40S-40S interface of collided ribosomes and promote ubiquitination of specific 40S proteins and the ASC complex that disassembles the leading collided ribosome (16). This then triggers downstream quality control events, including ribosome subunit splitting and recycling by ATP-binding cassette sub-family E member 1 (ABCE1) (17) and seemingly random C-terminal Ala and Thr addition (CAT-tailing) of stalled nascent peptides (NPCs) before their turnover by the ubiquitin-proteasome system (18). ABCE1 is required for several phases of translation, including ribosome recycling, translation re-initiation, and ribosome biogenesis (19). While our knowledge of the players and mechanisms involved in RQC continues to grow, there is still much to uncover about how cellular signaling pathways regulate the RQC process.

The abnormalities in translation observed in cancer cells, such as the reading through of stop codons, shifting of reading frames, and the stalling of ribosomes due to oxidative stress, indicate a potential involvement of the RQC pathway (20, 21). To better understand the role of RQC factors in cancer biology, more in-depth mechanistic studies are needed. Furthermore, the dysregulation of RQC factor expression in cancer cells, including that of ASCC3, ANKZF1, ABCE1, and VCP, highlights the intricate nature of their roles in cancer (22–25). It is noteworthy that these factors, and sometimes even the same factor, can have opposite effects in promoting oncogenesis or tumor suppression under different conditions (15), suggesting context-dependent effects of RQC in cancer.

The role of mitochondrial RQC has not been documented in cancer cells thus far. Interestingly, it was shown that mitochondrial damage blocks the cotranslational import of mitochondrial proteins and leads to the recruitment of RQC factors in neurodegenerative disease models (26). In this study, we investigated the function of ABCE1 using patient-derived primary cultured cancer cell models. We found that ABCE1 participates in the quality control of MOM-associated translation of mitochondrial proteins by the cytosolic ribosomes and ABCE1 interacts with electron transport chain (ETC) complex-I protein C-I30. Translational stalling induces mitochondrial translocation and aggregation of RQC factors ABCE1, ZNF598, and NEMF and interaction of ABCE1 with mitochondrial UPR protein HSP60, suggesting activation of mitochondrial unfolded protein response (mito-UPR). This stalling-induced mitochondrial stress (SIMS) may cause caspase-dependent cell death in cultured cancer cells and cancer stem cells and *in vivo* in a *Drosophila* brain tumor model. ABCE1’s role as a stabilizer of mitochondrial function during translational stalling highlights its previously unexplored potential in mitigating mitochondrial stress in cancer cells. Harnessing the mitochondrial translocation of ABCE1 and other RQC factors could potentially open new avenues for anti-cancer therapies.

## RESULTS

### Translational inhibition by EME induces alterations in mitochondrial physiology and function

**Figure 1.**
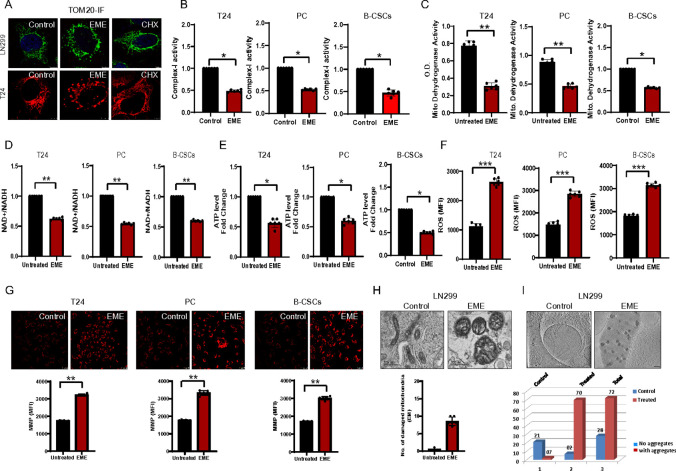
Translational inhibition by EME induces alteration in mitochondrial physiology and functions. (A) TOM20 staining in T24 bladder cancer and LN299 glioblastoma cells after emetine (EME, 50μg) and cycloheximide (CHX, 50μg) 4h treatment. (B) Complex I activity in T24, bladder cancer primary culture cells, and patient-derived bladder tumor spheroids after 4h of EME treatment. (C) Mitochondrial dehydrogenase activity in T24, bladder cancer primary culture cells, and patient-derived bladder tumor spheroids in the presence or absence of EME (50μg). (D) NAD+/NADH ratio in T24, bladder cancer primary culture cells, and patient-derived bladder tumor spheroids. (E)ATP level in T24, bladder cancer primary culture cells, and patient-derived bladder tumor spheroids. (F) ROS levels in T24, bladder cancer primary culture cells, and patient-derived bladder tumor spheroids. (G) MMP levels were measured by TMRM staining using flow cytometry in T24, bladder cancer primary culture cells, and patient-derived bladder tumor spheroids. (H) Transmission electron microscopy micrographs of untreated and EME (4h, 50μg)-treated mitochondrial fraction from LN299 cells. (I) Cryo-ET micrographs of untreated and EME-treated (4h, 50μg) mitochondria from LN299 cells.

Extensive research has highlighted the increasing role of ribosomes in cancer progression, leading to investigations into potential therapeutic interventions targeting ribosomes (27). While significant work has been done on using translation inhibitors for cancer treatment, there are limited studies that define the fate of ribosomes under these conditions. RQC is an essential process that maintains the integrity of the cellular proteome. Since mitochondrial dysfunction and proteostasis failure frequently coexist in cancer, it is crucial to understand the function of RQC proteins in cancer. To investigate the role of RQC in cancer, we used emetine (EME), a known translational inhibitor that can cause ribosome stalling and collision (PMID: 30293783). We found that EME induced donut-shaped mitochondrial morphology in both LN299 glioblastoma cells and T24 bladder cancer cells (Fig. 1A). The effect of EME on mitochondrial physiology was further investigated. We observed decreased mitochondrial ETC complex-I activity in bladder cancer cell line (T24), patient-derived muscle-invasive bladder cancer primary culture cells (PC), and patient tissue-derived tumor spheroids (Fig. 1B). In addition, we found that EME treatment led to significant inhibition of mitochondrial dehydrogenase activity in T24, PC, and tumor spheroids compared to untreated cells (Fig. 1C). Moreover, NAD^+^/NADH ratio was found to be decreased (Fig. 1D), as was ATP level (Fig. 1E) after EME treatment compared to untreated cells. Validation of physiological changes in mitochondria was studied by investigating the status of ROS and mitochondrial membrane potential (MMP). EME treatment resulted in a significant increase in ROS production (Fig. 1F) and hyperpolarization of mitochondria (Fig. 1G) in T24, PC, and tumor spheroids compared to untreated cells. The effect of EME on mitochondrial physiology was also assessed in the kidney cancer cell line A498, melanoma cell line A375, and cervical cancer cell line HeLa **(Suppl. Fig. S1)**. MMP plays an important role in protein import into mitochondria (28). Our transmission electron microscopy shows mitochondrial changes during EME treatment. The majority of mitochondria in the untreated cells have organized tubular cristae (left panels), whereas mitochondria in the EME-treated glioblastoma cells appeared damaged with a marked loss of cristae and the remaining cristae looked vesicular or swollen (Fig. 1H). Further cryo-electron tomography (cryo-ET) of mitochondria isolated from glioblastoma cells revealed the presence of large electron-dense mitochondrial granule clusters (MGCs) (~ 50 to ~ 100 nm size) positioned closely to the cristae regions (Fig. 1I). The untreated glioblastoma cells (control group) showed a low percentage of MGCs (about 20 to 25%; n = 28) in isolated mitochondria while, strikingly, the EME treatment group showed MGCs in 97% mitochondria (n = 72). These Cryo-ET studies and supporting biochemical data strongly suggest that the mitochondrial aggregation (MGC formation) phenomenon is tightly coupled to mitochondrial dysfunction caused by the translation inhibitor EME.

### Translation arrest by EME recruits the RQC factors to mitochondria

**Figure 2:**
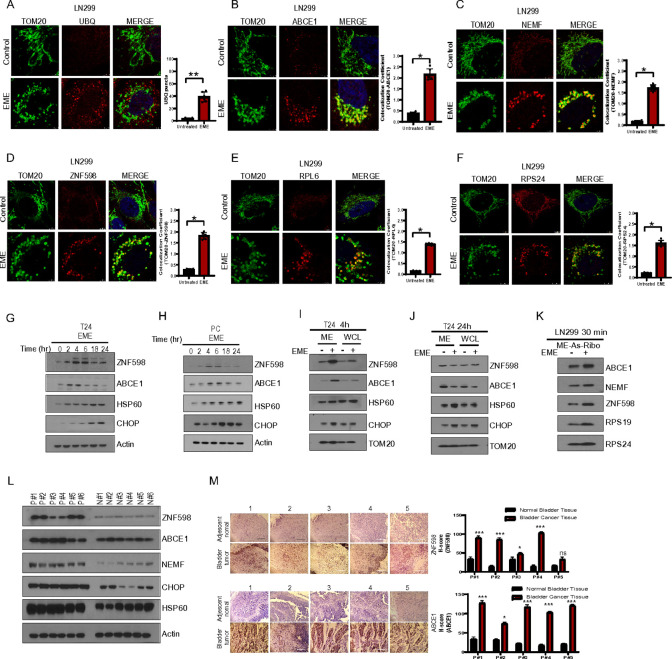
Translation arrest by EME recruits RQC factors to mitochondria. (A) TOM20 (green) and Ubiquitin (UBQ; red) immunostaining in EME-treated (4h, 50μg) LN299 cells. Images were collected using confocal microscope at 63X, zoom-4. (B) TOM20 (green) and ABCE1 (red) immunostaining in LN299 cells. (C) TOM20 (green) and NEMF (red) immunostaining in LN299 cells. (D) TOM20 (green) and ZNF598 (red) immunostaining in LN299 cells. (E, F) TOM20 (green) and RPL6 (red) immunostaining (E), and TOM20 (green) and RPS24 (red) immunostaining (F) in LN299 cells. (G, H) Western blot images of indicated proteins after EME (50μg) treatment in T24 (G) or PC cells (H). (I, J) Western blot images of RQC proteins in the mitochondrial fraction (ME) and whole cell lysates (WCL) of T24 cells at 4h (I) and 24h (J) treatment with EME. (K) Western blot images showing the expression of RQC proteins in mitochondria-associated ribosomes in EME-treated LN299 cells. (L) Western blot images showed RQC protein status in patients (#P) and normal (#N) bladder muscle invasive high-grade bladder tumor-tissue samples (N=5). (M) Microscopic images of IFHC show ABCE1 and ZNF598 expression in normal bladder tissues and bladder tumor tissues.

Our data showed that EME increases the MMP and leads to loss of inner membrane integrity. This may be caused by the increased burden of incomplete translation products. Previous studies have documented the dynamic ubiquitination of a variety of ribosomal proteins, suggesting that ubiquitylation plays a critical role in regulating ribosomal function during the RQC process (29). Our cryo-EM data revealed the presence of aggregated MGCs during EME treatment, prompting an investigation into the nature of these aggregates by assessing ubiquitin levels. EME treatment increased the accumulation of ubiquitin on the mitochondria (as shown by TOM20 and ubiquitin colocalization) compared to untreated LN299 glioblastoma cells (Fig. 2A). Increased ubiquitination on the mitochondrial surface is one of the indicators of translational stalling (11). Therefore, we checked RQC factor colocalization with the mitochondrial marker TOM20. EME treatment led to increased colocalization of ABCE1 (Fig. 2B), NEMF (Fig. 2C), and ZNF598 (Fig. 2D) with TOM20. In addition, EME treatment increased the colocalization of RPL6 (Fig. 2E) and RPS24 (Fig. 2F) with TOM20. RQC protein translocation to mitochondria was further validated by the colocalization of ABCE1 and NEMF with mitochondrial ETC chain complex-I protein (CI-30). Our immunostaining data showed colocalization of these RQC factors with CI-30 by EME treatment **(Fig. S2A, B)**. To determine the specificity of mitochondrial localization of these RQC factors during EME-mediated translational stalling, we investigated additional ribosomal quality control proteins such as ERF1 and PELO (11). We did not observe significant colonization of ERF1 or PELO with TOM20 **(Fig.S2C)** We next dissected the kinetics of RQC protein involvement. EME treatment led to increased levels of RQC proteins ZNF598 and ABCE1 at early time points in both T24 and primary cultured cancer cells (Fig. 2G, H). Mitochondrial proteostasis response is managed by mitochondrial unfolded protein response (mt-UPR). The expression of mt-UPR markers CHOP (30) and HSP60 (31) was upregulated by EME treatment at later time points. Translocation of the RQC factors to mitochondria was further investigated in mitochondrial subcellular fraction. RQC protein accumulation was observed at the 4h time point, whereas mito-UPR proteins were upregulated at 24h in the mitochondrial fraction (Fig. 2I, J). EME-induced RQC factor enrichment was also detected in the ribosomal fraction isolated from mitochondria (Fig. 2K).

To correlate these *in vitro* findings with patient data, we collected bladder tumor tissues (n = 6) and adjacent normal bladder tissue (n = 6). Interestingly, the expression of RQC protein and mito-UPR response proteins was higher in bladder cancer patient tissues than in normal bladder tissues (Fig. 2L). This implicated a role for RQC in cancer progression. This finding was also corroborated by the IHC study in patient samples. For the IHC study, a total of five muscle-invasive high-grade bladder cancer patient tissues were collected after radical cystectomy. As controls, adjacent normal bladder tissues were collected under the supervision of a consulting surgeon. A significantly higher expression of ABCE1 and ZNF598 was found in tumor tissues compared to adjacent normal tissues (Fig. 2M). These findings support a potential role of RQC factors in facilitating tumor progression.

### Entrapment of stalled translation products in the mitochondrial matrix results in mitochondrial dysfunction

**Figure 3:**
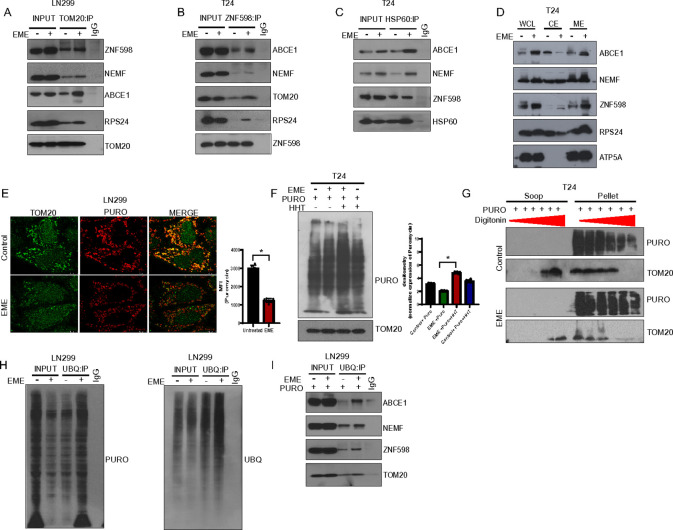
Entrapment of unresolved translated products in the mitochondrial matrix results in mitochondrial dysfunction. (A) Western blot images showing TOM20 co-immunoprecipitation (co-IP) in mitochondrial fraction in LN299 cells. (B, C) Western blot images showing ZNF598-colP (B) and FISP60 co-IP (C) using the mitochondrial fractions of EME-treated T24 cells. (D) Western blot images showing RQC factor translocation from the cytosol to mitochondria during EME treatment in T24 cells. (E) Labelling of NPCs: Immunostaining of TOM20 (green) and puromycin (red) in the presence or absence of EME in LN299 cells. (F) Western blot images showing the level of puromycin incorporation during HHT and EME treatment in T24 cells. Bar graph shows increased incorporation of puromycin by EME and HHT treatment. (G) Mitochondrial membrane permeabilization with increasing concentrations of digitonin. EME-treated cells showed resistance of puromycin positive signals representing stalled NPCs to digitonin treatment. (H, I) Western blot analysis of ubiquitin immunoprecipitation (UBQ-IP) in cells with or without EME and HHT treatment.

The accumulation of RQC factors in mitochondria was further investigated using a Tom20 immuno-pulldown assay. This revealed increased TOM20 interaction with ABCE1, ZNF598, NEMF, and RPS24 by EME treatment (Fig. 3A). This interaction was validated by reverse ZNF598 immunoprecipitation. Moreover, increased ZNF598 interaction with ABCE1, NEMF, RPS24, and TOM20 was observed after EME treatment (Fig. 3B). To test if RQC factor interaction inside mitochondria is directly involved in EME-induced mitochondrial dysfunction and mito-UPR response, we did co-IP with the mito-UPR protein HSP60 and found increased interaction with ZNF598, ABCE1, and NEMF by EME treatment (Fig. 3C). This result suggests that EME induced translational stalling leads to RQC protein entrapment in mitochondria, causing SIMS and triggering the mito-UPR response to cope with stress.

RQC factors are found at substoichiometry levels relative to the ribosomal proteins (31, 32). This scarcity of RQC factors poses a challenge under stress conditions when ribosome collisions increase. We were next interested in exploring RQC factor distribution among different subcellular compartments. The addition of EME resulted in reduced ABCE1 level in the cytosol and increased ABCE1 level in the mitochondrial fraction (Fig. 3D). The level of NEMF did not significantly change in the cytosolic fraction but increased mitochondrial level of NEMF was observed upon EME treatment. ZNF598 level was increased in both the cytosol and mitochondria, however stronger enrichment of ZNF598 was observed in the mitochondrial fraction after EME treatment (Fig. 3D). To further test the specific localization of RQC factors inside mitochondria, we performed protease protection analysis upon disruption of the outer membrane by hypotonic swelling. Signals for RQC factor ABCE1, ZNF598, and NEMF were preserved under this condition, demonstrating their localization inside mitochondria (Fig.S3A).

To test whether the proper handling of NPCs associated with stalled translation is altered by EME, puromycin labeling was conducted in the presence of HHT, which inhibits new translation but allows active ribosomes to run off (33). EME/HHT cotreatment led to the accumulation of puromycin incorporation compared to HHT alone (Fig. 3E, F). This may reflect EME induced CAT tailing of stalled translation products. These observations also raised the question of whether EME induces NPC entrapment inside mitochondria. To test this possibility, we treated the mitochondrial fraction with digitonin at different doses to permeabilize mitochondrial outer membrane. Strikingly, the puromycin signals in EME-treated mitochondrial fraction were more resistance to digitonin treatment compared to untreated cells, suggesting the matrix localization of EME-induced signals (Fig. 3G). Stalled NPCs are known to be ubiquitinated for degradation under normal conditions. We next performed ubiquitin pulldown under puromycin labeling conditions with or without EME treatment. Our western blot analysis showed increased ubiquitinated NPCs and associated RQC factors by EME treatment (Fig. 3H, I). NPC entrapment was further confirmed by hydroxylamine (HA) treatment, which releases NPCs by attacking peptidyl-tRNA bond (34). Microscopic analysis showed that EME-induced aggregation of NPCs was inhibited by HA treatment **(Fig. S3B)**. These results suggest that in the presence of EME, protein translation is halted, which further drives the continued import of stalled RQC complex inside mitochondria.

### Translocation of RQC factors from the cytosol to mitochondria promotes cancer cell death

**Figure 4:**
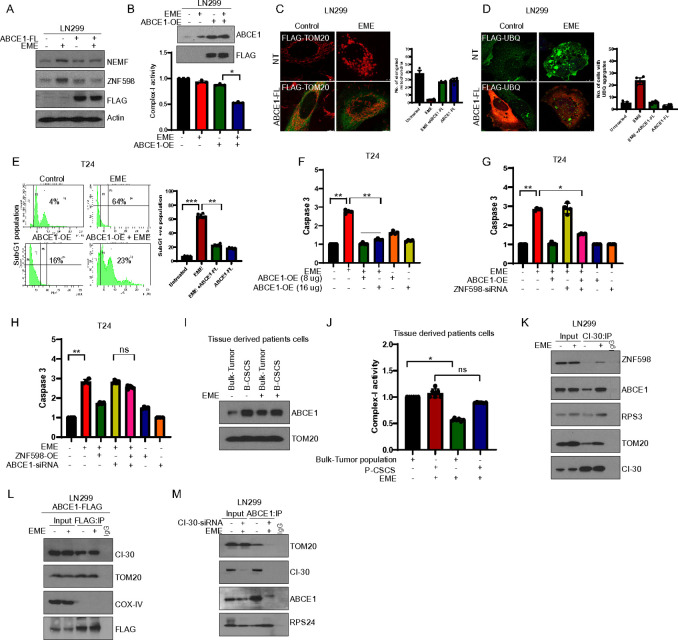
Translocation of RQC factors from the cytosol to mitochondria promotes cancer cell death. (A) Western blot images showing RQC protein expression in ABCE1-FLAG overexpressing LN299 cells in the presence of EME at4h. (B) Bar graph showing complex-l activity in EME-treated ABCE1 overexpressing LN299 cells. The upper panel shows western blot analysis of the transfection efficacy of ABCE1 overexpression. (C, D) TOM20 (red)-FLAG (green) immunostaining (C)and UBQ (green)-FLAG (red) immunostaining (D) in EME-treated ABCE1 overexpressing LN299 cells. (E) SubG1 analysis of EME-treated ABCE1 overexpressing T24 cells. (F) Bar graph showing caspase-3 activity in ABCE1-expressing cells in the presence of EME. (G) T24 cells were treated with ZNF598 siRNA followed by ABCE1-FLAG overexpression. The bar graph shows caspase-3 activity in EME-treated ABCE1 cells deficient for ZNF598. (H) T24 cells were treated with ABCE1-siRNA, followed by ZNF598-FLAG overexpression. The bar graph shows caspase-3 activity in EME-treated ZNF598 overexpressing T24 cells deficient for ABCE1. (I) Western blot showing ABCE1 expression in the mitochondrial fractions of bulk T24 cells and tumor tissue-derived cancer stem cells (B-CSCs) treated with EME. (J) Bar graph showing complex-l activity in the EME-treated bulk cancer population and bladder cancer stem cell population. (K) C-I30 immunoprecipitation in LN299 cells. Western blot shows C-I30 and RQC protein interaction in EME-treated cells. (L) Immunoprecipitation of FLAG in ABCE1-FLAG overexpressing LN299 cells. Western blot shows ABCE1 interaction with mETC proteins. (M)ABCE1 immunoprecipitation in C-I30 silenced cells. Western blot shows C-I30 mediated ABCE1 interaction with TOM20 in EME-treated LN299 cells.

Given the known roles of ABCE1 and ZNF598 in the RQC pathways, we consider the possibility that ABCE1 and ZNF598 cooperate in protecting mitochondrial protein homeostasis and function under translational stalling conditions. To test the role of ABCE1 in mitochondrial homeostasis, we overexpressed an ABCE1-FLAG plasmid in LN299 glioblastoma cells. EME-induced accumulation of RQC factors such as ZNF598 and NEMF was rescued by ABCE1 overexpression (Fig. 4A). Interestingly, ABCE1 overexpression significantly restored ETC complex-I activity during EME treatment (Fig. 4B). In addition, ABCE1 overexpression rescued EME-induced mitochondrial morphology changes as evidenced by TOM20 immunofluorescence (Fig. 4C). Ubiquitin accumulation induced by EME was also rescued in ABCE1 overexpressing LN299 cells (Fig. 4D). To further confirm that ABCE1 plays a role in maintaining mitochondrial function, we overexpressed ABCE1 in a concentration-dependent manner. Interestingly, we found that higher expression of ABCE1 was restoring more mitochondrial complex-I activity during EME treatment **(Fig. S4A)**.

EME treatment led to caspase activation in cancer cells. We next asked whether ABCE1 was capable of rescuing this EME effect. Significant blockage of EME-induced caspase activation by ABCE1 expression was observed in subG1 population analysis by flow cytometry (Fig. 4E) and caspase-3 activity assay (Fig. 4F). These results suggest that continuous and prolonged SIMS can make ABCE1 a limiting factor that causes cancer cell apoptosis when its level is below a threshold. However, cells with higher ABCE1 levels may escape SIMS-induced cell death. To investigate the involvement of other RQC factors, we overexpressed ZNF598 and checked the mitochondrial function. ZNF598 overexpression rescued mitochondrial complex-I **(Fig. S4B)** and caspase-3 activity **(Fig. S4C)** in EME-treated cells. Combined overexpression of ABCE1 and ZNF598 conferred greater rescue of complex-I and caspase-3 activity under EME treatment compared to ABCE1 or ZNF598 overexpression alone **(Fig. S4D, E)**. Given ZNF598’s proposed function in protecting mitochondria under stress conditions (35), we hypothesized that ABCE1 and ZNF598 work together to maintain mitochondria protein homeostasis. We assessed caspase-3 activity in the absence of ZNF598 under ABCE1 overexpression condition. EME-induced caspase-3 activity was significantly rescued by ABCE1 overexpression in ZNF598 silenced cells (Fig. 4G) On the contrary, ZNF598 overexpression was not able to rescue caspase-3 activity in the absence of ABCE1 (Fig. 4H), suggesting a vital role of ABCE1 in mitochondrial homeostasis during translational stalling and an epistatic relationship between ABCE1 and ZNF598 in this process.

The above findings indicate that EME treatment causes an excessive build-up of RQC proteins within mitochondria, disrupting mitochondrial proteostasis and ultimately triggering cell death. ABCE1 localization into mitochondria was further confirmed in complex-I protein C-I30 colocalization studies. ABCE1 was found to be colocalized with C-I30, however, ABCE1 colocalization was not observed with ETC complex-IV protein Cox-IV (Fig. F4F). ABCE1 expression was further investigated in tumor–tissue derived bulk cancer population and cancer stem cell population. Interestingly, the expression of ABCE1 was higher in CSCs compared to the bulk tumor population (Fig. 4I) EME treatment decreased the complex-I activity of cancer cells, however, no significant effect of EME was found on CSCs (Fig. 4J). These results suggest a potential role for ABCE1 in conferring resistance and recurrence in cancer cells. Further, C-I30 interaction with ABCE1 was validated by CI-30 co-IP (Fig. 4K). C-I30 interaction was also observed with ZNF598 and ribosomal proteins RPS3. These interactions were also confirmed in ABCE1 overexpressing cells (Fig. 4L). To test whether ABCE1 requires C-I30 for maintaining mitochondrial function, we silenced C-I30 and performed ABCE1 co-IP. Surprisingly, the absence of C-I30 led to decreased interaction between TOM20 and the RQC factors (Fig. 4M) As the translation of C-I30 is stalled by mitochondrial stress (11), these data suggest that ABCE1 is a rate-limiting factor during the RQC of stalled translation. EME induces the translocation of ABCE1 from the cytosol to mitochondria. This results in the cytosol having less ABCE1 for the quality control of ribosome function, ultimately leading to cancer cell death.

### Increased MMP leads to aggregation of RQC proteins inside mitochondria

**Figure 5:**
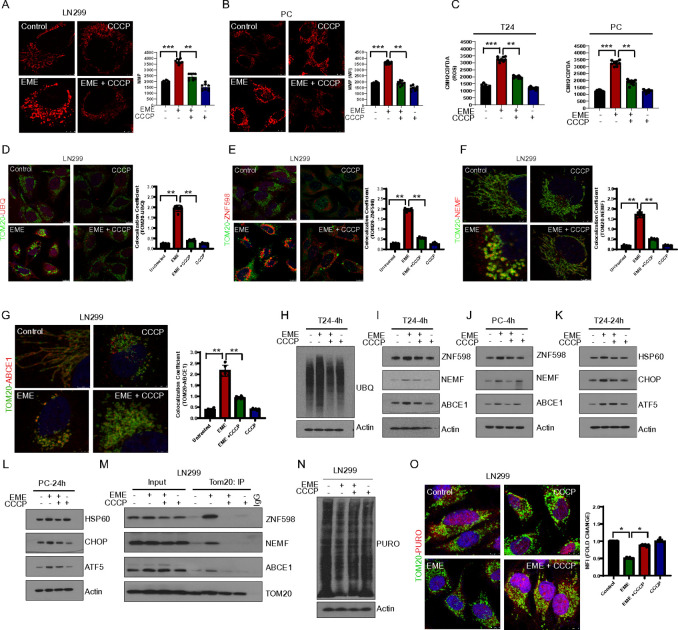
Increased MMP leads to aggregation of RQC proteins in mitochondria. (A, B) Confocal images of TMRM staining after EME (50μg) and CCCP (2.5) treatment in LN299 cells (A) and patients-derived primary culture (PC) cells (B). (C) Quantification of CM-H2DCFDA staining in patients derived primary culture a nd T24 cells. (D) TOM20 (green) and UBQ (red) immunostaining in LN299 cells with EME and CCCP co-treatment (4h). (E-G) TOM20 (green)-ZNF598 (red) immunostaining (E), TOM20 (green)-NEMF (red) immunostaining (F), TOM20 (green)-ABCE1 (red) immunostaining (G) in LN299 cells with EME and CCCP co-treatment. (H-J) Western blot images showing UBQ and RQC protein status in T24 (H, I) and primary culture cells (J). (K, L) Western blot images showing RQC proteins status at 24h treatment with EME and CCCP in T24 (K) and primary culture cells (L). (M) TOM20 immunoprecipitation in the mitochondrial fraction of EME and CCCP treated LN299 cells. (N) Immunoblots show ubiquitin levels in LN299 cells under EME and CCCP treatment condition at 4h. (O) Immunostaining of TQM20 (green) and puromycin (red) in LN299 cells under EME and CCCP treatment condition at 4h.

One of the striking effects of EME is increased MMP (Fig. 1G). Intact MMP is key to protein import (36). We asked whether by decreasing MMP we could rescue the EME effect on mitochondrial physiology. To verify this, we checked MMP using TMRM staining in the presence of a very low dose of a mitochondrial uncoupling agent CCCP. Interestingly, CCCP treatment rescued the EME-induced hyperpolarization in LN299 (Fig. 5A) and primary culture cells (Fig. 5B) EME-induced ROS was also rescued by CCCP treatment (Fig. 5C). EME induced accumulation of ubiquitin inside mitochondria was also restored by CCCP (Fig. 5D). Mitochondrial translocation of the RQC proteins ABCE1, NEMF, and ZNF598 was also blocked by CCCP treatment (Fig. 5E–G). Global ubiquitination induced by EME was also inhibited by CCCP (Fig. 5H). The levels of RQC proteins ZNF598, NEMF, and ABCE1 induced by EME was decreased by co-treatment with CCCP in T24 and primary culture bladder cancer cells (Fig. 5I, J). We next examined the effect of CCCP on mito-UPR proteins. EME-induced upregulation of mitochondrial UPR response proteins CHOP, ATF5, and HSP60 was restored by CCCP treatment (Fig. 5K, L). Interaction of TOM20 with RQC factors ZNF598, NEMF, and ABCE1 was also inhibited by CCCP co-treatment in EME-treated cells (Fig. 5M). Moreover, EME-induced stalled translation was rescued by CCCP treatment (Fig. 5N). This was further validated by puromycin immunostaining of stalled NPCs (Fig. 5O) These data suggest that an EME-induced hyperpolarization leads to a burden of unresolved translational products. Hyperpolarization has also been reported to induce autophagy. To test the possible involvement of autophagy, an autophagy inhibitor chloroquine (CQ) was administered. In contrast to CCCP, CQ treatment did not rescue the EME effect on mitochondrial morphological changes and ubiquitination level **(Fig. S5A, B)**. These results suggest that protein aggregation during EME treatment depends on mitochondrial membrane potential, affecting critical quality control machinery for mitochondrial homeostasis.

### Functional significance of EME induced SIMS on cancer cell fate

**Figure 6:**
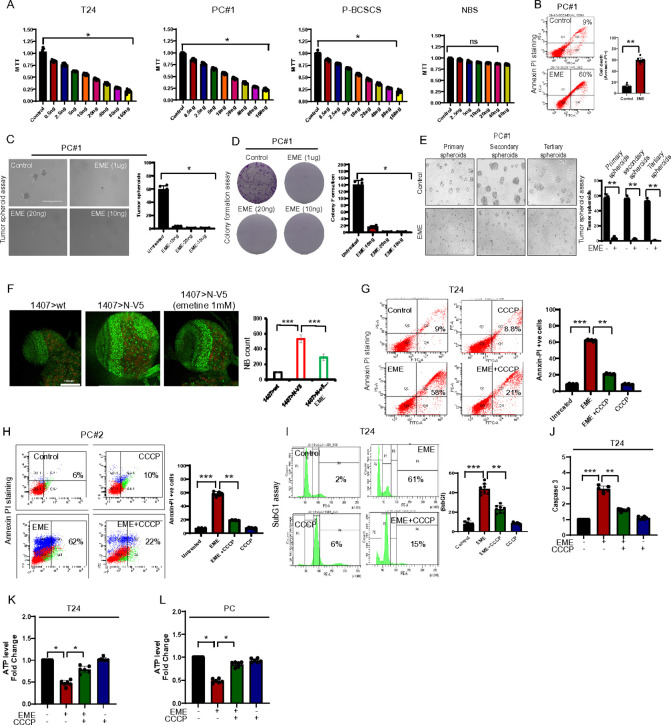
Functional significance of translational inhibitor on cancer cell fate. (A) Bar graphs showing MTT cell viability assays in T24, primary culture cells (PC#1), patient-derived spheroids, and normal bladder cells (NBC). (B) FACS data of Annexin-PI staining in primary culture cells. The bar graph shows an increased cell death population in EME treatment in primary culture bladder cancer cells. (C) Tumor spheroid assay in primary culture bladder cancer cells. Different concentration of EME was added to the tumor spheroids. The bar graph shows the decreased number of tumor spheroids in the presence of EME. (D) Colony formation assay in primary culture cells: Cells were treated with different concentrations of EME. The bar graph shows decreased colonies in the presence of EME. (E) Images showing primary cultured bladder cancer cell-derived primary, secondary, and tertiary tumor spheroids. (F) Effects of EME on NB number in Notch OE larval brain. Brains were immunostained for the NB marker Deadpan (green) and the differentiation marker Prospero (red). Bar graph shows data quantification. (G, H) FACS data of Annexin-PI staining in T24 (G) and primary culture bladder cancer cells (H). (I) FACS data for SubG1 analysis in T24 cells. (J) Bar graph showing colorimetric caspase-3 activity assay data in T24 cells in the presence of EME and CCCP for 24h. (K, L) ATP assay using colorimetric method in T24 (K) and primary culture bladder cancer cells (L) treated with EME and CCCP for 24h.

We next evaluated the functional implications of our findings of EME induced SIMS in cancer cells. The effect of EME on cell viability was investigated in a panel of different cancer cell lines. The cytotoxic effect of EME was found to be significantly higher in LN299, T24, primary bladder cancer cells, bladder cancer stem cells, HeLa, A375, and A549 cells compared to normal bladder cells (Fig. 6A; **Fig. S6A)**. The effect of EME on cell survival was further validated by Annexin-V/PI staining (Fig. 6B). Tumor spheroid formation was also decreased by EME treatment even at nanomolar concentration (Fig. 6C) Colony formation assay further validated EME potency in causing cancer cell death (Fig. 6D). We next established Tissue-Derived Tumor Spheres for studying the effect of EME on tumor spheroid formation. Treatment of EME significantly decreased the number of primary spheroids, secondary spheroids, and tertiary spheroids (Fig. 6E). To test the physiological relevance of EME effect on cancer growth *in vivo*, we first used a *Drosophila* brain tumor model induced by Notch OE driven by the neural stem cell specific 1407-Gal4 driver (37). We found that Notch-induced brain tumors exhibited a high number of neuroblasts compared to control brains (Fig. 6F). Treatment with EME significantly reduced the number of neuroblasts (Fig. 6F).

Since CCCP was shown to rescue the EME effect on mitochondrial physiology, we wanted to know whether CCCP could also abrogate EME-induced cell death in cancer cells. The impact of EME on cell death was reverted by CCCP treatment in T24 and primary culture cells (Fig. 6G, H). The subG1 population analysis also confirmed that EME-induced apoptosis was rescued by CCCP treatment (Fig. 6I). CCCP inhibition of EME effect on caspase-3 activity and ATP level was also confirmed in T24 and PC cells (Fig. 6J–L). Morphological studies further confirmed CCCP rescue of EME effect on cell death **(Fig. S6B, C)**.

## DISCUSSION

We have shown that translational stalling caused by EME leads to increased aggregation of RQC factors and NPCs inside mitochondria and decreased mitochondrial function. Upon import into mitochondria, these NPCs and RQC factors aggregate and sequester chaperones and proteases, eventually leading to proteostasis failure, respiratory deficiency, and breakdown of mitochondrial function (i.e., SIMS). ABCE1 functions to circumvent this SIMS and thus can be defined as an RQC component specifically adapted to curtailing the detrimental effects of stalled polypeptides that undergo mitochondrial translocation. Stalled NPCs with CAT tails are prone to aggregation. By restricting stalling, ABCE1 facilitates the passage of less aggregation-prone proteins into the mitochondrial matrix that can be adequately dealt with by the intra-mitochondrial quality-control system.

Cancer cells demand an enhanced protein production machinery, particularly in the form of ribosomes, to support their rapid growth and division. This increased demand for protein synthesis is driven by the need for more efficient housekeeping proteins and other cellular components that are required for unchecked cell division (38). As a result, ribosomes have long been considered as a possible target for cancer therapy. The abnormal production and altered function of ribosomes are observed in various types of cancers. This highlights the central role of ribosome biogenesis and protein synthesis as critical and limiting factors in the uncontrolled growth and proliferation of cancer cells, providing potential targets for therapeutic interventions in oncology. Here, we have shown that translational stalling induced by EME caused an increase in ROS level, hyperpolarization of MMP, and decreased complex-I activity. Dissipation of MMP by CCCP attenuates the import of RQC proteins and aggregation of NPCs inside the mitochondria. Although MMP dissipation normally results in reduced ATP levels and heightened caspase activity and eventual cell death, in the context of EME treatment, CCCP protects against cancer cell death. This supports the notion that EME induced toxicity was caused by the import of aberrant stalled translation products into mitochondria and subsequent perturbation of mitochondrial protein homeostasis.

Maintaining the balance of proteins in the mitochondria is vital for cellular health and has implications for various human diseases. This balance is primarily regulated by a network of mitochondrial chaperones and proteases. When proteomic imbalance or mitochondrial dysfunctions occur, cells initiate protective mechanisms to repair and maintain organelle integrity (39). Cancer cells have been observed to have unusually high mitochondrial membrane potential (ΔΨm) (40). However, further investigation is needed to understand whether this trait is caused by inherent mutations specific to cancer cells or by the activation of abnormal internal signaling pathways. Determining the sources of altered ΔΨm in cancer cells could aid in identifying new targets to combat cancer cells by limiting their metabolic flexibility and possibly their capacity to metastasize. Given that unusually high ΔΨm has been observed in a wide range of epithelial carcinoma cells (40), understanding the mechanisms that give rise to this trait could lead to the development of new therapeutic targets for various types of carcinomas. In our current study, we have found that when translational stalling is induced by EME, it causes the MMP to hyperpolarize. Interestingly, we have observed a direct correlation between the high MMP and steady-state internalization of ABCE1 into mitochondria, causing a reduction of ABCE1 levels in the cytosol. Consequently, this process leads to the internalization of other RQC factors such as ZNF598 and NEMF, and the NPCs presumably produced by cytosolic ribosomes that are known to be associated with the mitochondrial outer membrane (11, 41). Subsequently, these internalized proteins form aggregate within the mitochondria, prompting the activation of mitochondrial unfolded protein response and initiation of caspase-dependent cell death. The internalization of stalled NPCs and RQC factors inside mitochondria is consistent with the previous observation in yeast of a role of mitochondria as a backup destination for aberrant proteins when the cytosolic protein quality control mechanisms are overwhelmed (42).

**Figure 7:**
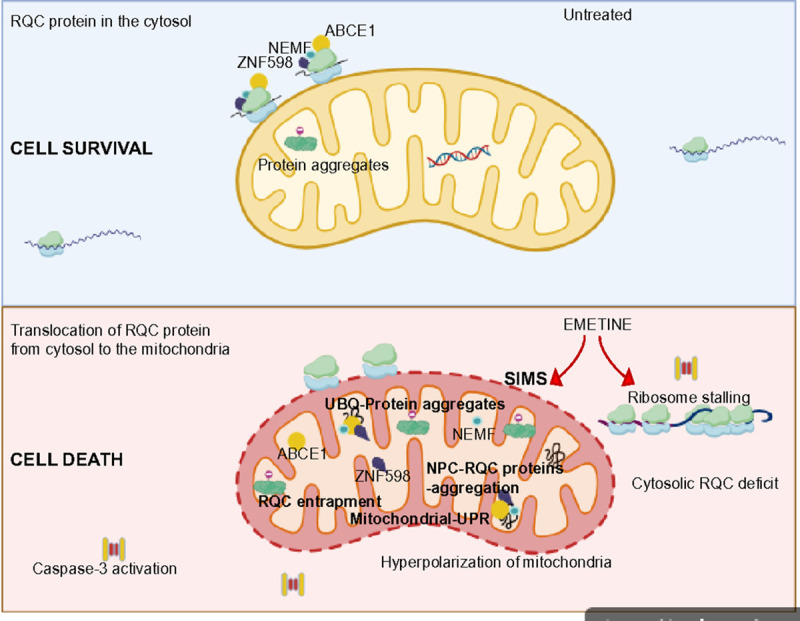
Model of SIMS induction of cancer cell death that involves RQC protein accumulation inside mitochondria. Under non-stress conditions, RQC proteins are present in the cytosol and maintain increased protein synthesis demands of cancer cells. EME induces SIMS that involves the translocation of ABCE1, ZNF598, and NEMF from the cytosol to mitochondria. This translocation is coupled with entrapment of NPC and associated RQC factors inside mitochondria, leading to activation of mitochondrial unfolded protein response. The entrapment of RQC factors inside mitochondria also leads to cytosolic RQC deficit. These events ultimately lead to the activation of caspase 3 and cell death.

ABCE1 is a member of the ATP-binding cassette superfamily. ABCE1 is a complex protein with intriguing mechanisms of action (43). Over the past two decades, there has been significant focus on understanding the biological roles and regulation of ABCE1. However, there is still much work needed to comprehend how ABCE1 contributes to tumor formation and metastasis. It is anticipated that further research into the biological regulation of ABCE1 could validate it as a potential therapeutic target for various types of cancers. In our studies, we found a high expression of ABCE1 in muscle-invasive high-grade bladder cancer tissues compared to normal bladder tissues. In addition, primary bladder cancer cells and patient-derived glioblastoma cells showed high expression of ABCE1. These results suggest ABCE1’s potential role in metastasis. Treatment with EME resulted in increased translocation of ABCE1 inside mitochondria. This led to reduced availability of ABCE1 in the cytosol, further disrupting the substoichiometry of RQC machinery in cancer cell cytosol. The mitochondrial translocation of ABCE1 along with other RQC factors increased the burden of stalled translation products and aggregated proteins inside mitochondria, causing a disturbance in mitochondrial proteostasis. This SIMS ultimately results in the death of cancer cells (Fig. 7). Our results assign a key role to ABCE1 in protecting mitochondria against the toxic effects of translational stalling. Furthermore, ABCE1 overexpression limits the cytotoxic effects induced by EME, providing insights into how ABCE1 hinders the impact of drugs in cancer cells. ABCE1 is recognized as a marker of drug resistance and plays a crucial role in the survival of cancer cells (44). The present study revealed ABCE1’s dual effect on cancer cell fate during translational stalling. Its cytoplasmic abundance leads to cell survival, while its mitochondrial translocation and aggregation causes cancer cell death (Fig. 7). The interaction of ABCE1 with the mETC complex-I protein C-I30 suggests possible additional roles of ABCE1 in maintaining mitochondrial function. This is consistent with the mitochondrial localization of ABCE1 in *Drosophila* and mammalian cells under normal conditions (45, 46). Further studies are necessary to confirm this unique role of ABCE1. In addition, a new binding partner, HSP60, has been identified for ABCE1 in this study, supporting its role in maintaining mitochondrial protein homeostasis. Thus, further research is warranted to dissect the exact roles of ABCE1 in cancer cells.

Emetine is regarded as a possible anti-cancer therapy for a variety of human tumors because of its ability to induce apoptosis. Emetine has been shown to inhibit cell survival, migration, and ultimately invasion in multiple cancers (47). The drug has been studied in conjunction with other drugs to offer synergistic antitumor effects for achieving successful therapy with a lower dose and fewer side effects (48). It has been clinically demonstrated that emetine inhibits the Hedgehog signaling pathway by binding to its constituent upstream and downstream signaling proteins like Hedgehog, Smoothened, and Gli, all of which have been associated with CSC biology (49). In the present study, we have highlighted the unique mechanism of action of EME in the context of effecting cancer cell death. EME causes translational stalling which leads to ABCE1 translocation from the cytosol to mitochondria. This translocation leads to the induction of mitochondrial unfolded protein response markers which ultimately leads to caspase-dependent cell death. Our findings suggest the promising prospects that emetine may augment the anticancer efficacy of specific chemotherapy drugs in various cancers, e.g. bladder cancer, glioblastoma, melanoma, and renal cell carcinoma.

## CONCLUSION

The significance of RQC factors in maintaining the quality of translation, especially MOM-associated translation, is a new area of investigation that is highly relevant to cancer biology. It is imperative to rectify any defect in these pathways, as they could be involved in the development and progression of various pathologies associated with mitochondria malfunction. ABCE1’ role as a stabilizer of mitochondrial function during translational stalling highlights its previously underexplored potential in mitigating mitochondrial stress. Furthermore, harnessing the mitochondrial translocation of ABCE1 and other RQC factors could potentially open new avenues for the therapeutic intervention of cancer and possibly other diseases.

## MATERIALs AND METHODS

**Table:** 

REAGENT or RESOURCE	IDENTIFIER	SOURCE
**Antibodies**		
Mouse anti-FLAG	F1804	Sigma-Aldrich
Rabbit anti-FLAG	F7425	Sigma-Aldrich
Mouse anti-TOM20	sc17764	Santa Cruz Biotech
Rabbit anti-C-I30	Ab110246	Abcam
Mouse anti-C-IV	ab14705	Abcam
Mouse anti-Actin	A2228	Sigma-Aldrich
Rabbit-ABCE1	28548-1-AP	Proteintech
Rabbit-ZNF598	A305-108A	Bethyl Laboratories
Rabbit-ubiquitin	10201-2-AP	Proteintech
Rabbit-HSP60	PAA822Ga01	Cloudclone
Rabbit-NEMF	11840-1-AP	Proteintech
Rabbit-eRF1	ab153731	Abcam
Rabbit-PELO	10582-1-AP	Proteintech
Mouse-puromycin	11840-1-AP	Sigma
Goat anti-Mouse IgG-HRP	sc-2005	Santa Cruz
Goat anti-Rabbit IgG HRP	sc-2004	Santa Cruz
Goat anti-mouse IgG (H+L) Highly Cross-Adsorbed Secondary Antibody, Alexa Fluor 488	A32723	Invitrogen
Goat anti-Rabbit IgG (H+L) Highly Cross-Adsorbed Secondary Antibody, Alexa Fluor 594	A11036	Invitrogen
DAPI	D9542	Sigma-Aldrich
**Chemicals, Peptides, and Recombinant Proteins**		
Emetine	E2375	Sigma-Aldrich
CCCP	C2759	Sigma-Aldrich
Cycloheximide	S7418	Selleckchem
Puromycin	ant-pr-1	Invivo Gen
Chloroquine	C6628	Sigma-Aldrich
Lipofectamine 2000	11668019	Invitrogen
Lipofectamine RNAi-MAX	13778150	Invitrogen
Tris base	11814273001	Sigma-Aldrich
Glycine	G8898	Sigma-Aldrich
SDS	L3771	Sigma-Aldrich
Pierce 16% Formaldehyde (w/v), Methanol-free	28908	ThermoFisher
Triton^™^ X-100	T9284	Sigma Aldrich
DMEM, high glucose, GlutaMAX Supplement	10566016	GIBCO
RPMI-1640	11875085	ThermoFisher
EDTA	E9884	Sigma-Aldrich
EGTA	E3889	Sigma-Aldrich
Dimethyl sulfoxide	D8418	Sigma-Aldrich
4XLaemmli sample buffer	161-0747	BioRad
Complete^™^, Mini, EDTA-free Protease Inhibitor Cocktail	11836170001	Roche
**Critical Commercial Assays**		
Complex I Enzyme Activity Microplate Assay Kit	ab109721	Abcam
NAD+/NADH quantification colorimetric kit	K337	BioVision
ATP assay kit	MAK190	Sigma-Aldrich
Caspase-3 assay kit	CASP3C	Sigma-Aldrich
Western Lightning Plus-ECL	NEL105001EA	PerkinElmer Inc.
HyBlot CL Autoradiography Film	1159M38	Denville Scientific Inc.
Tetramethylrhodamine (TMRM)	T668	Invitrogen
Di(Acetoxymethyl Ester) (6-Carboxy-2’,7’-Dichlorodihydrofluorescein Diacetate)	C2938	Thermo Fisher Scientific
NuPAGE^®^ MOPS SDS running buffer	NP0001	Invitrogen
NuPAGE 4–12% Bis-Tris Protein Gels	NP0321	Invitrogen
**Si-RNA**		
Stealth RNAi^™^ siRNA of NDUFS3	Invitrogen	HSS116992
Stealth RNAi^™^ siRNA of ABCE1	Invitrogen	HSS10925
Stealth RNAi^™^ siRNA of ZNF598	Invitrogen	149321
**Recombinant DNA**		
FLAG-ABCE1	Sino Biological	HG15502-G
FLAG-ZNF598	Addgene	105690
**Software and Algorithms**		
GraphPad Prism	GraphPad Software	
FlowJo	FlowJo	http://www.flowjo.com/
Excel	Microsoft	
Fiji ImageJ	National Institute of Health	
BioRender		https://www.biorender.com/
**Experimental Models: Cell Lines**		
HEK293 cell line	ATCC	CRC-1573
HeLa cell line	ATCC	CCL-2
A375 cell line	ATCC	CRL-1619
LN299 cell line	ATCC	CRL-2611
T24 cell line	ATCC	HTB-4
A498 cell line	NCCS	
GBM-387	Stanford University	SU-GBM002/387

### METHOD DETAILS

#### Sample size:

We have recruited fifty patients with 90% power of study and 95% confidence interval. The calculation was done using G*Power software version 3.1.9.4. The patients who met the study’s criteria and consented were recruited over a period of one and a half years (July 2022 to December 2023) after undergoing Radical cystectomy surgery. Each patient provided a complete clinical history and underwent thorough examinations, including all necessary investigations to confirm and stage their bladder tumor, using cystoscopy and histopathology report (HPR). The protocol was approved by the institute ethics committee, PGIMER (PGI/IEC/2024/EICO00330; PGI/IEC/2023/ 0006, IEC-01/2024–2984). All histopathologically confirmed cases of high-grade muscle-invasive bladder cancer were recruited in the study. Patients with a history of other malignancies, a history of radiotherapy and chemotherapy, or bladder perforation were not included in the study. Each experiment was repeated in triplicates. For primary culture, N=20 patient samples were collected. For, IHC, N=5 patients’ tumors and adjacent normal bladder tissue were used. For western blot experiments in tissue samples, N=6 patients’ tumors and adjacent normal bladder tissue were used. The rest of the patient’s samples were used in the PGIMER Bio-banking facility for further research work.

#### Cell culture and cell transfection conditions

Regular LN299, A375, HeLa, HEK293, and T24 cells were obtained from the American Tissue Culture Collection (ATCC) and were maintained under standard tissue culture conditions (5% CO2, 37°C). SU-GBM002/387 cell line was obtained from Drs. Sid Mitra and Sam Checher who generated it from a freshly resected human GBM sample acquired under Stanford University School of Medicine approved IRB protocol.

Primary culture of bladder cancer cells (N=20) was established from the tissues of patients with muscle-invasive high-grade bladder cancer. Adjacent normal bladder tissue (N=20) was collected from the same MIBC-high-grade bladder cancer after complete resection of the bladder under the supervision of the consultant in charge. Primary bladder cancer cells and normal bladder cells were cultured in DMEM high glucose containing 10% FBS. To characterize bladder cells cytokeratin-7 staining was performed using FACS (data not shown). For experiments, cells from passage 3 were used.

Cell transfections were performed by using Lipofectamine 2000, and siRNA knockdown experiments were performed using Lipofectamine RNAiMAX reagent, according to the manufacturer’s instructions.

#### Tissue-derived spheroids formation for isolating bladder cancer stem cells

For the generation of tumor-derived spheroids, tumor tissues were collected from bladder tumor patients after radical cystectomy. Tumor tissue was carried to the lab in a DMEM medium containing antibiotics. Tissue was minced with a scalpel blade and then crushed with a striated plunger from a disposable syringe in a BSL2 hood. The resulting pieces were seeded in ultralow attachment in KSFM medium with a growth supplement.

#### Immunofluorescence analysis of cultured cells

For immunofluorescence analysis, cells were washed with 1x PBS three times and fixed with 4% formaldehyde in 1x PBS for 30 min at room temperature, later washed and permeabilized with 1x PBS containing 0.1% Triton X-100 for 5 min. The fixed samples were subsequently blocked with 1x PBS containing 5% normal goat serum and incubated for 1 hour at room temperature followed by incubation with primary antibodies for 3 hrs at room temperature. Thereafter, secondary antibodies were added for 1 hour at room temperature. The primary antibodies used were mouse anti-TOM20, ABCE1, NEMF, ZNF598, Puromycin, PELO, (1:1,000, Santa Cruz). The secondary antibodies used were Alexa Fluor^®^ 488 and 594 conjugated antibodies (1:500, Molecular Probes).

#### Mitochondria isolation

Intact mitochondria from human cells were purified and quality controlled for the absence of contamination by other organelles according to the established procedures. Briefly, samples were homogenized using a Dounce homogenizer. After two steps of centrifugation (1,500 g for 5 min and 13,000 g for 17 minutes), the mitochondria pellet was resuspended and washed twice with HBS buffer (5 mM HEPES, 70 mM sucrose, 210 mM Mannitol, 1 mM EGTA, 1x protease inhibitor cocktail). The mitochondrial pellet was resuspended in appropriate buffers for further analysis. Each experiment was conducted in triplicate and repeated three times.

#### Mitochondria-associated ribosome isolation

For isolation of mitoribosomes, the mitochondrial pellet was resuspended in ribosome isolation buffer A with 10% NP40 for 10 min followed by centrifugation at 1000g for 10 minutes. The supernatant was collected and centrifuged at 10000g for 12 minutes at 4°C. The supernatant was collected and 0.2M KCl was added. Thereafter, the supernatant was applied to an ultracentrifuge tube containing 1M sucrose cushion. The centrifugation was done at 40000 rpm for 1h at 4°C. The pellet was resuspended in 1x protein sample buffer for western blot analysis.

#### Mitochondrial respiratory chain complex I activity assay

Mitochondrial complex-I activities were measured by using a Complex I Enzyme Activity Microplate Assay Kit (Colorimetric). Briefly, cells were seeded and mitochondrial extracts were prepared and quantified by using the Bradford reagent. Mitochondrial extracts were added to coated plates and incubated for 3 hrs. After incubation, wells were washed with wash buffer three times. Thereafter, wells were blocked with a blocking buffer for 1 hour. After blocking, wells were washed with wash buffer, and assay buffer was added for 30 min. After incubation, O.D. was observed at 450 nm using a microplate reader (Cytation3, BioTek Inc). Each experiment was conducted in triplicate and repeated for three times.

#### Co-immunoprecipitation (co-IP), SDS-PAGE, and western blot analyses

Cell lysates were processed directly in NP40 IP-lysis buffer (5 M NaCl, 10% NP-40, 1 M Tris (pH 8.0), with protease inhibitor cocktail added. After centrifugation at 10,000 g for 5 min, the supernatant was subjected to incubation with primary antibodies at 4°C overnight with gentle shaking. Subsequently, the magnetic beads were added for 2 hrs and thereafter washed three times (10 minutes each) at 4°C in a washing buffer. 1x loading dye was added and samples were boiled at 97°C for 5 min. Samples were loaded onto NuPAGE 4%–12% Bis-Tris Protein Gels and ran in MOPS SDS running buffer and immunoblot analyses according to standard procedures. For data quantification of western blots, signal intensity was measured and calculated using NIH Image J.

#### Flow cytometric analysis of ROS

Cells were seeded at 0.5×10^6^ cells/ml. Cells were treated with various drugs for 30 min to 4 hrs and thereafter, cells were trypsinized and processed for staining with CM-H2DCFDA (5μM). Cells were incubated with CM-H2DCFDA for 15 min in the dark at 37°C. After incubation, cells were washed and resuspended in PBS. Samples were immediately observed using an LSR II flow cytometer, and data were analyzed using the FlowJo software.

#### ROS measurement by CM-H2DCFDA with a microplate reader

5×10^4^ cells were seeded and treated as indicated in figures. After treatment, cells were trypsinized and stained with CM-H2DCFDA (5μM) for 15 min in the dark at 37°C. After incubation, cells were washed and resuspended in DPBS. Stained cells were added in a black-walled, clear-bottom 96-well. Emission was observed at 492–495/517–527 nm for 30 min at 37°C on a Multi-Mode Reader (BioTek Instruments). Each experiment was repeated three times.

#### Mitochondrial membrane permeability measurement by TMRM

2×10^5^ cells were seeded and treated on cell culture plates as indicated in figures. After treatment, cells were trypsinized and stained with TMRM (20nM) for 15 min in the dark at 37°C. After incubation, cells were washed and resuspended in DPBS. Stained cells were added in a black-walled, clear-bottom 96-well. Emission was observed at 574 nm on a Multi-Mode Reader (BioTek Instruments). Each experiment was repeated three times.

#### NAD^+^/NADH measurement

NAD^+^/NADH was assayed using an NAD^+^/NADH quantification colorimetric kit (Amplite^®^ Colorimetric NAD/NADH Ratio Assay Kit) according to the manufacturer’s instructions. Briefly, 2×10^5^ cells were seeded and treated with EME (50ng) for 4 hrs. After treatment, cells were pellet by centrifuging at 2,000 rpm for 5 min. Cells were incubated with lysis buffer for 15 min at 4°C and cell lysates were collected after centrifugation at 12,000 g for 15 min. 100μl samples were added to 96-well plate. For NADH measurement, the NADH reaction mixture was added to the well and incubated at 37°C for 15 min and absorbance was observed at 460 nm. For the measurement of the total NAD^+^/NADH amount, NAD extraction solution was added into the lysates and incubated at 37°C for 15 min, thereafter neutralization solution was added to neutralize the NAD extracts. Absorbance was monitored at 460 nm. The ratio of NAD^+^/NADH was determined by the following equation: ratio = NAD (total) − NADH/NADH.

#### ATP measurement

ATP level was measured with the ATP fluorometric Assay Kit (Sigma) by following the manufacturer’s protocol. Briefly, 2×10^5^ cells were seeded and treated with EME with or without CCCP (2.5μM) for 24 h. After treatment, cells were lysed in an ATP assay buffer. Thereafter, the ATP probe, ATP converter, and developer mix were added sequentially in a black bottom 96 well plate. ATP standards were used to identify the samples/unknown values. After addition of the buffer in their respective places, the plate was incubated for 30 min in the dark and thereafter took the reader at 535/ 587 nm.

#### Caspase 3 activity assay

Caspase 3 activity was measured by using the Caspase 3 Assay Kit, Colorimetric (Sigma; CASP3C) according to the manufacturer’s protocol. Briefly, 2×10^5^ cells were seeded and treated with EME (50μg) and CCCP (2.5μM) for 24h. After treatment, 1× Assay Buffer was added to a flat bottom black 96-well plate. Thereafter, the caspase 3 inhibitor was added to the appropriate well followed by the addition of caspase 3 substrate for 90 minutes of incubation at 37°C in the dark. Caspase 3 activity was measured using a multimode reader at 408 nm.

#### Cytotoxicity assay

Annexin and propidium iodide (Annexin-V/PI) dyes were used for cell death assays. Protocols were followed as per the manufacturer’s instructions. Briefly, 3×10^6^ cells were seeded and treated with EME (50μg) for 24 hrs. After treatment, cells were trypsinized and washed with PBS. Cell pellets were stained with Annexin-PI for 30 min in the dark at 37°C. After incubation, cells were immediately observed using a flow cytometer and data was analyzed by using the FlowJo software. Each experiment was conducted in duplicated and repeated three times.

#### SubG1 analysis

For SubG1 analysis, 2×10^5^ cells were seeded and treated with EME for 24 h. After incubation, cells were fixed in 70% ethanol for overnight. Thereafter, cells were incubated with RNAse buffer for 30 minutes followed by 30 minutes of PI incubation in the dark at 37°C. Cells were immediately analyzed by flow cytometry.

#### Fluorescence/morphological examination

The effect of emetine on cell morphology was investigated by staining cells with a combination of fluorescent DNA binding dyes AO/EB (Acridine Orange/Ethidium Bromide) as described previously (50). Briefly, cells were treated with different concentrations of EME for 24 h and cell viability was determined under Invitrogen fluorescent microscope. For analysis of nuclear morphology, Hoechst staining was carried out under confocal microscope.

#### MTT assay

For MTT assays, cells were plated in 96-well plates (2,000 cells/well), and allowed to adhere overnight, The Next day, cells were treated with EME (0.5–160ng) for 72 hrs. After treatment, MTT (5mg/ml) was added and incubated for 4 hrs. Thereafter, MTT solubilizing buffer was added and O.D. was observed after 30 min at 570 nm. Data was analyzed using Microsoft Excel and statistical significance was calculated by using Graphpad. Each experiment was repeated three times.

#### Clonogenicity assay

For colony formation assay, primary culture cells and T24 cells were plated in 96-well plates (500 cells/well), and allowed to adhere overnight, The next day, cells were treated with EME (10ng, 20ng, and 1μg), or DMSO vehicle control for two weeks. After treatment, cells were washed with PBS and crystal violet solution (0.5% crystal violet solution in 25% methanol) was added for 15 min at room temperature. After incubation, the plates were washed with water. After drying the plates, colonies were scanned and quantified in Adobe Photoshop (Adobe Photoshop CC2017). Each experiment was repeated three times.

#### Tumor sphere formation assay

To test for the effect of EME on GBM-387 CSCs and bladder cancer-derived tumor spheroids, 200 cells/well were plated in a 96-well plate and treated with EME or DMSO vehicle control. Spheroids were observed for one to two weeks. The number of spheroids formed was counted under an inverted microscope and graphs were plotted using GraphPad. Each experiment was repeated three times.

#### Secondary and tertiary sphere formation assay

To examine the ability of tumor spheres to form the next spheroid generations, spheroids were harvested, treated with trypsin/EDTA, and mechanically dissociated by gently pipetting. The resulting single cells, after counting, were re-plated in serum-free spheroid medium (KSFM) at the same densities and culture conditions as mentioned above for three sequential passage times.

#### Protease protection assay

3×10^6^ A375 cells were seeded overnight. The next day, cells were scrapped and mitochondrial extracts were prepared using a Dounce homogenizer in HBS buffer (5 mM HEPES, 70 mM Sucrose, 210 mM Mannitol, 1 mM EGTA, 1x protease inhibitor cocktail). 50 μg mitochondrial extracts were treated with digitonin (2%) and different doses of trypsin (0–2.5μg) for 30 min on ice. Thereafter, centrifugation was done at 10,000×g for 15min. The mitochondrial pellets were used for western blot analysis. Tom20 was used as markers for mitochondrial outer membrane. C-I30 was used as an inner membrane marker.

#### Puromycin labeling of ribosome stalled newly synthesized proteins

3×10^6^ T24 cells seeded on 10 cm^2^ dishes were incubated with fresh DMEM medium. Cells were treated with EME (100μg) for 4h. After incubation, cells were first treated with HHT (5 μM for 10 min at 37 °C) followed by Puromycin (100 μM) addition to the medium, and cells were incubated further for 15 min at 37 °C. Cells were then washed and harvested in a lysis buffer, and processed for western blot analysis and immunoprecipitation.

Puromycin labeling of stalled proteins: Puromycin labeling was done as described previously. LN299 cells were seeded on coverslips in a 6-well plate and treated with EME (100 μg) for 4h. After incubation, cells were treated with HHT for 5 min, after that Puromycin (50 μg) was added and cells were incubated for 5 min. Once the incubation was over, cells were permeabilized by 0.02% digitonin in Permeabilization buffer (50 mM Tris–HCl, pH7.5, 5 mM MgCl2, 25 mM KCl, 355 mM cyclohexamide, 10 units RNAseOut and 0.02% digitonin) for 2 min. Permeabilized cells were washed twice with washing buffer (permeabilization buffer without digitonin) and fixed in 4% paraformaldehyde for 30 min. The permeabilization and washing steps were performed in ice-old buffers. Cells were then stained with TOM20 and Puromycin antibodies and observed under the confocal microscope.

#### Immunohistochemistry (IHC)

Tumor tissue and normal bladder tissue were collected from patients with MIBC-high-grade bladder cancer after complete resection of the bladder with consent from the patients. Tumor tissue and normal bladder tissue were immediately fixed into formalin and processed for IHC staining in the histopathology department, PGIMER. IHC data were scored blinded by a pathologist (Nandita Kakkar).

Immunohistochemical stains were interpreted semiquantitatively (H-Score) by assessing the intensity and extent of staining on the entire tissue sections on the slides using a four-tiered (0 to 3) scale. First, the total percentage of positively stained tumor cells was determined. Then the percentage of weakly (1), moderately (2), and strongly (3) stained cells was determined so that the sum of these categories was equated with the overall percentage of positivity. A staining score was then calculated as follows: score (out of a maximum of 300) = the sum of 1 × percentage of weak, 2 × percentage of moderate, and 3 × percentage of strong staining.

#### Immunohistochemistry of fly brain tissues

For larval brain immunostaining, larvae were dissected in Schneider’s medium (Invitrogen) and fixed with 4% formaldehyde in PEM buffer (100 mM PIPES at pH 6.9, 1 mM EGTA, 1 mM MgCl2) for 23 min at room temperature. Immunohistochemistry with anti-Pros and anti-Dpn staining of *Drosophila* brain tumor samples was performed as described (37). The primary antibodies used were mouse anti-Pros (1:200) and guinea pig anti-Dpn (1:1000; J. Skeath). Corresponding secondary antibodies were from Molecular Probes (all at 1:200 dilution). Samples were imaged on a Leica SP8 confocal microscope and neuroblast numbers were counted and quantified.

#### Quantification and statistical analysis

Comparisons between two groups were calculated using one- or two-tailed Student’s *t*-tests, using GraphPad Prism software. Data are reported as mean ± SEM. Statistical values, including number of replicates (n), can be found in the figure legends. *p < 0.05, **p < 0.01, ***p < 0.001. For comparing multiple groups, we used a one-way ANOVA test followed by the Student–Newman–Keuls test (SNK test) plus Bonferroni correction (multiple hypotheses correction).

## STATEMENT OF SIGNIFICANCE

Our research found a strong link between RQC proteins ABCE1 and ZNF598 and high-grade bladder cancer. ABCE1 plays a key role in maintaining mitochondrial health during stalling-induced mitochondrial stress. Cancer stem cells with elevated ABCE1 expression show greater resistance to EME-induced mitochondrial dysfunction, indicating a potential mechanism of drug resistance. The pronounced effect of emetine on cancer cell death in both ex vivo and in vivo cancer models suggests its translational utility in the management of cancer.
